# Anti-Inflammatory and Anti-Thrombotic Effects of the Fungal Metabolite Galiellalactone in Apolipoprotein E-Deficient Mice

**DOI:** 10.1371/journal.pone.0130401

**Published:** 2015-06-15

**Authors:** Franziska Bollmann, Sven Jäckel, Lisa Schmidtke, Katharina Schrick, Christoph Reinhardt, Kerstin Jurk, Zhixiong Wu, Ning Xia, Huige Li, Gerhard Erkel, Ulrich Walter, Hartmut Kleinert, Andrea Pautz

**Affiliations:** 1 Department of Pharmacology, University Medical Center of the Johannes Gutenberg University Mainz, Mainz, Germany; 2 Center for Thrombosis and Hemostasis (CTH), University Medical Center of the Johannes Gutenberg University Mainz, Mainz, Germany; 3 Department of Molecular Biotechnology and Systems Biology, Technical University Kaiserslautern, Kaiserslautern, Germany; University of Leuven, BELGIUM

## Abstract

Patients suffering from chronic inflammatory diseases have an increased mortality risk resulting from cardiovascular disorders due to enhanced atherosclerotic and thrombotic events. Until now, it is not completely understood in which way an abnormal expression of pro-inflammatory mediators contributes to this elevated cardiovascular risk, but there is a need for new drugs that on the one hand suppress the expression of pro-inflammatory mediators and on the other hand inhibit arterial platelet adhesion. Thus, we analyzed the anti-inflammatory and anti-thrombotic capacity of the fungal metabolite Galiellalactone in atherosclerosis-prone apolipoprotein E-deficient mice. Treatment of the mice with Galiellalactone lowered the inflammatory expression profile and improved blood clotting times, as well as platelet adhesion to the injured common carotid artery. The results indicate that administration of Galiellalactone is able to reduce the extent of inflammation and arterial platelet adhesion in this mouse model.

## Introduction

Atherosclerosis as well as thrombotic events as its clinical manifestation results in increased cardiovascular death (about 50%) of people suffering from chronic inflammatory diseases, such as rheumatoid arthritis [1–4] or inflammatory bowel disease [5]. This increased mortality rate is independent of classic risk factors, as hypercholesterolemia, age, gender, or smoking [6]. Nowadays, the key role of inflammation during the progression of atherosclerosis is well accepted [7, 8]. Since pro-inflammatory mediators as tumor necrosis factor-α (TNFα) or Interleukin-1β (IL1β) are elevated in rheumatoid arthritis patients and trigger atherogenesis [9], the design of new drugs is focusing on the reduction of these inflammatory mediators. But so far, only contrary studies exist, demonstrating either beneficial or no beneficial effects of an anti-TNFα therapy on the cardiovascular risk of rheumatoid arthritis patients [10, 11]. Currently, there is a need for new drugs, which suppress vascular inflammation signaling to reduce atherosclerosis development and arterial thrombus formation.

Therefore, we investigated whether the fungal metabolite Galiellalactone (Gal) impairs inflammation and thrombosis in a model of Apolipoprotein E (ApoE)-deficient mice. In previous studies Gal was shown to be an anti-inflammatory compound that inhibits Interleukin-6 (IL6) signaling in a Janus Kinase/Signal Transducer and Activator of Transcription (STAT)—dependent manner and reduces experimental asthma [12, 13]. In addition, Gal has been described as a direct inhibitor of the transcription factor STAT3 [14] and to interfere with the nuclear import of the transcription factor nuclear factor kappa-light-chain-enhancer of activated B cells (NF-κB)[15].

For the first time, we now analyzed the anti-thrombotic capacity of this fungal metabolite with respect to inflammatory marker gene expression, blood clotting times, and *in vivo* platelet adhesion to injured carotid artery in mice.

Our results indicate a role for Gal in suppressing the expression of pro-inflammatory genes in the aorta, as well as inhibitory effects of Gal on the coagulation system and on platelet adhesion to the *Arteria carotis communis*.

## Materials and Methods

### Animals

ApoE-deficient mice (ApoE-ko) on a C57BL/6 background (stock 002052; The Jackson Laboratory) were housed in accordance with standard animal care requirements in groups up to five animals per cage. All mice were maintained under specific pathogen-free conditions on a 12/12 hours light/dark circle. Water and food were given *ad libitum*. Genotyping of the animals was performed by polymerase chain reaction, using primers that span the regions of the wild type genes disrupted by the targeting vectors. The following oligonucleotides (all obtained from Sigma-Aldrich; Hamburg, Germany) were used for genotyping the ApoE locus using polymerase chain reactions: ApoE-wt/for GCC TAG CCG AGG GAG AGC CG; ApoE-wt-rev TGT GAC TTG GGA GCT CTG CAG C; ApoE-ko-rev GCC GCC CCG ACT GCA TCT.

Experimental mice at the age of 6 weeks were either fed with normal chow diet (ND) or western-type diet (WD) for 18 weeks. For the last six weeks of this period, the WD-fed mice were treated every other day with an *intra peritoneal* injection of either the fungal metabolite Galiellalactone (Gal; 10 mg/kg) or vehicle (PBS + 10% ethanol; PBS/EtOH). All experiments were performed between 8 a.m. and 8 p.m. in the laboratory.

All procedures performed on mice were approved by the Institutional Animal Care and Use Committee (“Landesuntersuchungsamt” State of Rhineland-Palatinate, Germany; 23177-07/G11-1-010 and 23177-07/G11-1-018) in accordance with the German animal protection law and the guidelines for the use of experimental animals as stipulated by the Guide of Care and Use of Laboratory Animals of the National Institutes of Health. Mice were euthanized by *intra peritoneal* injection of 700 μL pentobarbital solution (1% pentobarbital in PBS), as not stated otherwise.

### Quantitative Real-Time Reverse Transcription Polymerase Chain Reaction Analyses

To analyze the gene expression, the RNA of aortas as well as livers from experimental animals was isolated. Therefore, the aortas or livers were homogenized in guanidinium thiocyanate buffer and total RNA was isolated by guanidinium thiocyanate/phenol/chloroform extraction as previously described [16].

For mRNA expression analyses, two-step real-time RT PCRs (qPCRs) using either TaqMan probes [17] or SYBR Green as described before [18] were performed. 500 ng total RNA was reverse transcribed using the High-Capacity cDNA Reverse Transcription Kit (Applied Biosystems; Darmstadt, Germany) following the manufacturer’s recommendations. Subsequently the qPCR reaction was performed using the following oligonucleotides (obtained from Sigma-Aldrich; Hamburg, Germany) as sense and antisense primers, as well as TaqMan hybridization probes (5’-6FAM; 3’-TAMRA) (Table 1).

**Table 1:** Oligonucleotides as sense and antisense primers and TaqMan hybridization probes.

CCL2/MCP1	Chemokine C-C motif ligand 2
Sense	AGG TCC CTG TCA TGC TTC TG
Antisense	TCA TTG GGA TCA TCT TGCT G
CTSS	Cathepsin S
Sense	CAT GGT GTT CTT GTG GTT GG
Antisense	CAA TAA CTA GCA ATT CCG CAG TG
Probe	CTG GCT TGT GAA AAA CAG TTG GGG C
GAPDH	Glyceraldehyde 3-phosphate dehydrogenase
Sense	TTC ACC ACC ATG GAG AAG GC
Antisense	GGC ATG GAC TGT GGT CAT GA
Probe	TGC ATC CTG CAC CAC CAA CTG CTT AG
FII	Coagulation factor II
Sense	CAG CTA TGA GGA GGC CTT TG
Antisense	TCA CAC CCA GAT CCA TAG CA
FVIII	Coagulation factor VIII
Sense	TGC CTG ACC CGC TAT TAT TC
Antisense	AGC GTT GCA TGT TCT CTG TG
FX	Coagulation factor X
Sense	TCA GCC TGC TCT GTG TTG TC
Antisense	TCG AAG ATT TCA CGG ACCTC
IL1β	Interleukin-1β
Sense	CAA CCA ACA AGT GAT ATT CTC CAT G
Antisense	GAT CCA CAC TCT CCA GCT GCA
IL6	Interleukin-6
Sense	GAG GAT ACC ACT CCC AAC AGA CC
Antisense	AAG TGC ATC ATC GTT GTT CAT ACA
Probe	TCC TAC CCC AAT TTC CAA TGC TCT CC
IL17	Interleukin-17
Sense	AGC AAG GAA TGT GGA TTC AGA G
Antisense	CAG AAA AAC AAA CAC GAA GCA G
Probe	TGC CCT CCA CAA TGA AAA GAA GGTG
iNOS	inducible nitric oxide synthase
Sense	CAG CTG GGC TGT ACA AAC CTT
Antisense	CAT TGG AAG TGA AGC GTT TCG
Probe	CGG GCA GCC TGT GAG ACC TTT GA
Pol2a	RNA polymerase 2a
Sense	ACC ACG TCC AAT GAT ATT GTG GAG
Antisense	ATG TCA TAG TGT CAC ACA GGA GCG
Probe	CTG GGC ATT GAG GCT GTG CGG AA
S100A8	Calgranulin A
Sense	CTC CGT CTT CAA GAC ATC GTT TG
Antisense	TCA TTC TTG TAG AGG GCA TGG TG
Probe	CAA TGC CGT CTG AAC TGG AGA AGG CC
SPP1	Osteopontin
Sense	GCT TGG CTT ATG GAC TGA GG
Antisense	CCT CAT CTG TGG CAT CAG G
Probe	TCA AAG TCT AGG AGT TTC CAG GTT TCT GAT GA
TNFα	Tumor necrosis factor-α
Sense	CAT CTT CTC AAA ATT CGA GTG ACA
Antisense	TGG GAG TAG ACA AGG TAC AAC CC
Probe	CAC GTC GTA GCA AAC CAC CAA GTG G

mRNA expression data were normalized either to Glyceraldehyde-3-phosphate dehydrogenase or DNA polymerase IIa mRNA expression. To calculate the relative mRNA expressions, the 2^(-ΔΔCt)^ method [19] was used.

### Coagulation Analyses

For coagulation analyses, freshly collected blood was incubated with 1/10 v/v citrate solution (Sigma-Aldrich; Hamburg, Germany) to inhibit blood coagulation. Afterwards, the blood was centrifuged for 20 minutes at 600 xg, the obtained plasma was diluted ¼ with PBS, and used for blood coagulation assays, using a KC4 Delta Coagulometer (Tcoag; Bray, Ireland).

To determine the prothrombin time (PT), 50 μL plasma was incubated for 1 minute at 37°C. After adding 100 μL thromborel S (Siemens Healthcare; Marburg, Germany) the time until complete clotting of the sample occurred was measured.

For analyzing the partial thromboplastin time (aPTT), 50 μL plasma was incubated with 50 μL pathromtin SL (Siemens Healthcare; Marburg, Germany) for 5 minutes at 37°C. The reaction was started after adding 50 μL of a 0.025 M calcium chloride solution (Siemens Healthcare; Marburg, Germany). The time until complete clotting of the sample occurred was measured.

### Hematologic Analysis

Citrated whole mouse blood was collected by intracardiac puncture. Platelet counts, white blood cell counts, and red blood cell counts were determined using an automatic cell counter (KX-21; Sysmex, Kobe, Japan).

### Mouse Thrombosis Model in the Common Carotid Artery

Experimental ApoE-deficient mice fed with ND or WD (treated with Gal or vehicle) were anesthetized by *intra peritoneal* injection of a solution of 5 mg/kg midazolame (Ratiopharm GmbH; Ulm, Germany), 0.5 mg/kg medetomidine (Pfizer Deutschland GmbH; Berlin, Germany), and 0.05 mg/kg fentanyl (Janssen-Cilag GmbH; Neuss, Germany), as described previously [20]. The animals were fixed on a custom-built stage and maintained at physiological temperature. As a model of arterial thrombosis, carotid injury was induced as previously described [21]. Therefore, a polyethylene catheter (0.28 mm ID, 0.61 mm OD; Smiths Medical Deutschland GmbH; Grasbrunn, Germany) was implanted into the right jugular vein. The left common carotid artery was dissected free and ligated vigorously (7.0 monofil polypropylene filament, Prolene; Ethicon; Norderstedt, Germany) near the carotid bifurcation for 5 min to induce vascular injury. Before and after vascular injury, the fluorescent platelets were visualized *in situ* by intra-vital epifluorescence high-speed video microscopy of the left common carotid artery. All mice with bleedings or any injury of the carotid artery during surgery were excluded from further analysis. There was no difference in the exclusion rate across the different experimental groups. All animals were euthanized by cervical dislocation.

### Preparation of Platelets for Intra-Vital Epifluorescence High Speed Video Microscopy

Citrated whole mouse blood was collected by *intra cardial* puncture. Murine platelets were isolated and labeled with 12.5 μg/mL 5-(and 6-) Carboxyflourescein diacetate, succinimidyl ester (Invitrogen Life Technologies GmbH; Carlsbad, California, USA) as reported earlier [20]. The labeled platelet suspension was adjusted to a final concentration of 150 x 10^3^ platelets/μL and 250 μL of the suspension was injected *intra venously via* a jugular vein catheter.

### Intra-Vital Epifluorescence High Speed Video Microscopy

Measurements were performed with a high-speed wide-field Olympus BX51WI fluorescence microscope, using a long-distance condenser and a 10x (NA 0.3) water immersion objective with a monochromator (MT 20E; Olympus Deutschland GmbH; Hamburg, Germany) and a charge-coupled device camera (ORCA-R2; Hamamatsu Photonics; Hamamatsu, Japan). For image acquisition and analysis, the Realtime Imaging System eXcellence RT software (Olympus Deutschland GmbH) was used. Cell recruitment was quantified in one field of view (100 μm x 150 μm) per injury area. Adherent cells were defined as cells that did not move or detach from the endothelial lining within an observation period of 20 seconds and presented per mm^2^.

### Isolation of Galiellalactone

Galiellalactone, a tetrahydro-isobenzofuranone derivative, from the ascomycete strain A111-95 was isolated as previously described [12].

The ascomycete strain A111-95 was isolated from wood. The strain was kindly provided by H. Anke and is deposited in the culture collection of the LB Biotechnologie, University Kaiserslautern. For maintenance on agar slants the strain was kept on YMG medium composed of: yeast extract 0.4%, malt extract 1%, glucose 1%, pH 5.5 and agar 1.5% for solid media. Fermentations were carried out in a Braun Biostat A-20 fermenter containing 20 liters of YMG medium with aeration (3 l air/ min) and agitation (120 rpm) at 22°C. The production of galiellalactone was followed by the inhibitory effect of various concentrations of a crude extract of the culture fluid in the IL-6-dependent reporter gene assays as described below. After 700 h of fermentation, the culture fluid was separated from the mycelium by filtration and extracted with EtOAc. The solvent was evaporated and the crude product (2.3 g) was separated by chromatography on silica gel (Merck 60) with cyclohexane:EtOAc (70:30) as eluent resulting in 1.2 g of an enriched product. Preparative HPLC (Macherey-Nagel Nucleosil 100–7 C-18, column 40U250 mm) with water:MeOH (46:54) as eluent yielded 635 mg galiellalactone. The purity of the isolated compound as estimated by HPLC-DAD/MS analysis was greater than 98.5%. Gal was resolved in ethanol abs. and administered at a concentration of 10 mg/kg in PBS containing 10% ethanol.

### Statistics

Data represent means + SEM, as not stated otherwise. Statistical differences were determined by factorial analysis of variance followed by one-way or two-way ANOVA multiple comparison test. In the case of two means, classic t-test analyses were used. All statistical analyses were performed using Graphpad Prism 5.0d.

## Results

### Galiellalactone Reduces Pro-Inflammatory Marker Gene Expression in Pro-Atherosclerotic Mice

**Fig 1:**
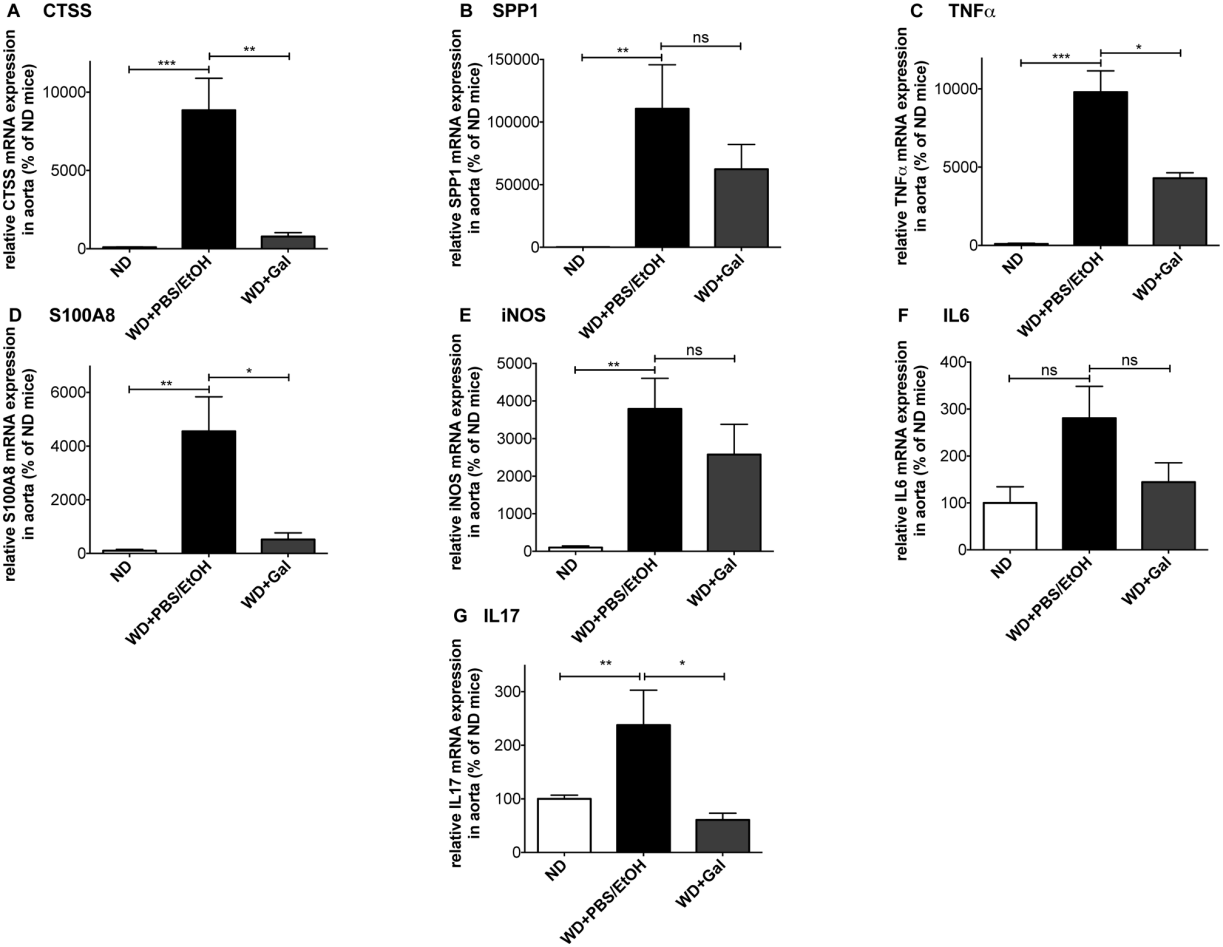
Galiellalactone reduces the expression of pro-inflammatory marker genes in pro-atherosclerotic mice. RNA isolated from aortas of ApoE-deficient mice either fed for 18 weeks with normal chow diet (ND) or western-type diet (WD) (treated with Galiellalactone (Gal) or vehicle (PBS/EtOH) for 6 weeks) was analyzed for CTSS (**A**), SPP1 (**B**), TNFα (**C**), S100A8 (**D**), iNOS (**E**), IL6 (**F**), and IL17 (**G**) mRNA expression in qPCR experiments. Data shown are mean + SEM of 4–10 mice (*** = p < 0.001; ** = p < 0.01; * = p < 0.05; ns = not significant vs. WD + PBS/EtOH; one-way ANOVA).

To test whether Gal-treatment results in reduced expression of pro-inflammatory genes in conductance arteries, we studied the expression profile of major pro-inflammatory mediators in the aorta of mice on WD that were either treated with vehicle (PBS+EtOH) or Gal. When compared with the ND group, feeding ApoE-deficient mice with WD for 18 weeks and treating them for 6 weeks with vehicle (PBS+EtOH) led to an increase of most analyzed marker gene mRNAs in the aorta that are known to have pro-atherosclerotic and/or pro-inflammatory capacities (Fig 1). These results demonstrate a highly inflamed and atherosclerosis-prone status in the aorta of WD- and vehicle-treated mice.

Gal-treatment of ApoE-deficient mice fed with WD for 18 weeks, decreased mRNA expression of the marker genes when compared to mice treated with vehicle. Gal-administration significantly reduced mRNA expression levels of pro-inflammatory TNFα (Fig 1C) and interleukin-17 (IL17) (Fig 1G), both published to be involved in atherosclerotic changes in ApoE-deficient mice [22, 23], Calgranulin A (S100A8) that is a marker for chronic inflammatory diseases (Fig 1D) [24], as well as the atherogenesis-related marker Cathepsin S (CTSS; Fig 1A) [25]. Furthermore, Gal-treatment decreased the mRNA-expression of other markers up to certain levels: Osteopontin (SPP1; Fig 1B), known to recruit monocytes/macrophages [26], inducible nitric oxide synthase (iNOS) that is critical for many immunomodulatory mechanisms (Fig 1E), as well as the pro-inflammatory cytokine IL6 (Fig 1F).

As no significant induction of IL1β and CCL2 (chemokine C-C motif ligand 2, also named monocyte chemotactic protein 1—MCP1) mRNA expression was detected in the same aorta RNA preparations, (see S1 Fig), we consequently measured no effect of Gal on the expression of these mRNAs.

### Treatment with Galiellalactone Leads to Prolonged Blood Clotting Times

To analyze whether Gal-treatment has functional effects on blood coagulation, which can induce platelet activation and is essential to stabilize nascent thrombi, we performed coagulation assays using plasma of ApoE-deficient mice fed with WD and treated either with vehicle or Gal.

**Fig 2:**
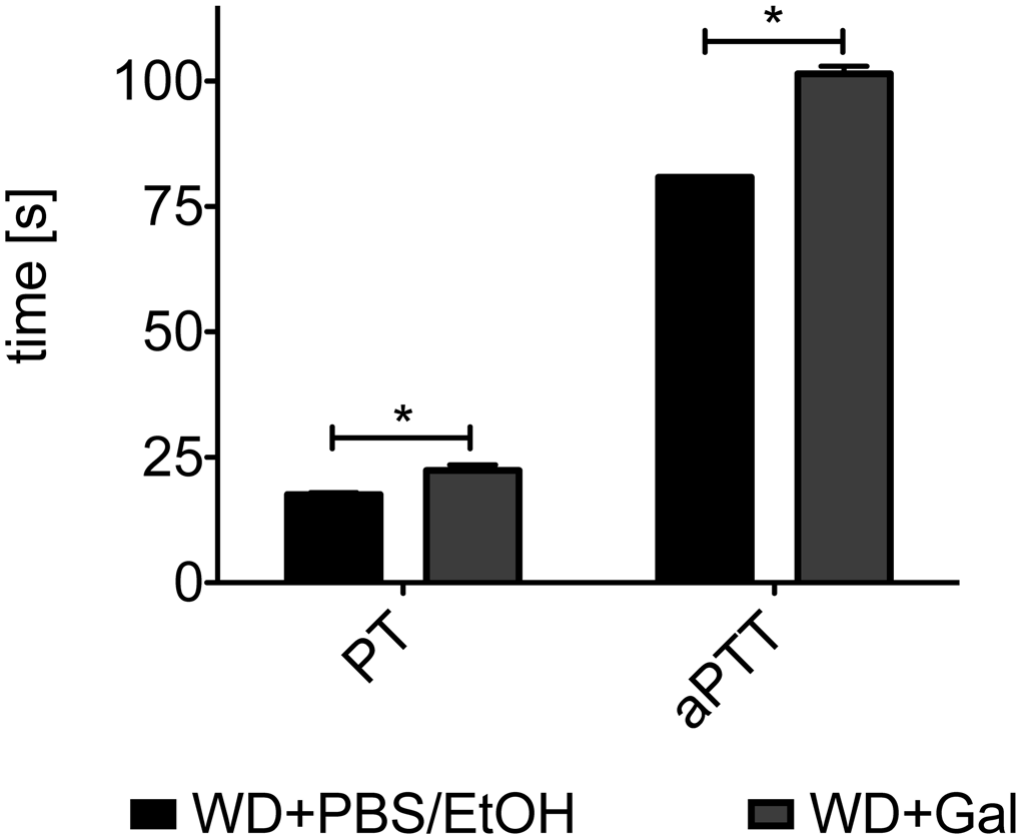
Galiellalactone-treatment of pro-atherosclerotic mice leads to prolonged blood clotting times. Citrate-buffered plasma of ApoE-deficient mice fed with WD for 18 weeks and treated either with PBS/EtOH or Gal for the last 6 weeks was used for blood clotting studies in a coagulometer. PT values were obtained by adding Thromborel S, whereas aPTT values were obtained by addition of Pathromtin SL and calcium chloride. The time until a complete clotting of the sample occurred was measured. Data shown are mean + SEM of 2–4 mice (* = p < 0.05 vs. PBS/EtOH-treated mice; t-test).

An every other day-treatment of mice with Gal over six weeks increases the time needed for complete coagulation of the samples (Fig 2). This increase was noted for both the intrinsic and the extrinsic pathway of the coagulation cascade, as PT values represent the extrinsic and aPTT values the intrinsic and common pathway of coagulation.

To check if these changes in blood clotting times are related to changes in the expression of clotting factors in the liver, we determined in qPCR analyses the mRNA expression of the clotting factors FII, FVIII and FX in the livers of the animals. As shown in S2 Fig, no effects of Gal on the mRNA expression of these factors could be detected.

### Galiellalactone Reduces Platelet Adhesion to Injured Arterial Blood Vessels

To analyze the anti-thrombotic capacity of Gal, we determined, whether a 6-week treatment of pro-atherosclerotic ApoE-deficient mice either fed with ND or WD (treated with Gal or vehicle) has an impact on platelet adhesion to the ligation-injured carotid artery, a vascular side where atherosclerotic plaques frequently develop and rupture. To monitor platelet adhesion over the time, a common carotid artery thrombosis model was used, in which fluorescence-labeled platelets isolated from a donor mouse of the same genotype and background were injected into the acceptor mouse [27]. Adhesion of fluorescent platelets was visualized *in situ* by intra-vital epifluorescence high-speed video microscopy.

**Fig 3:**
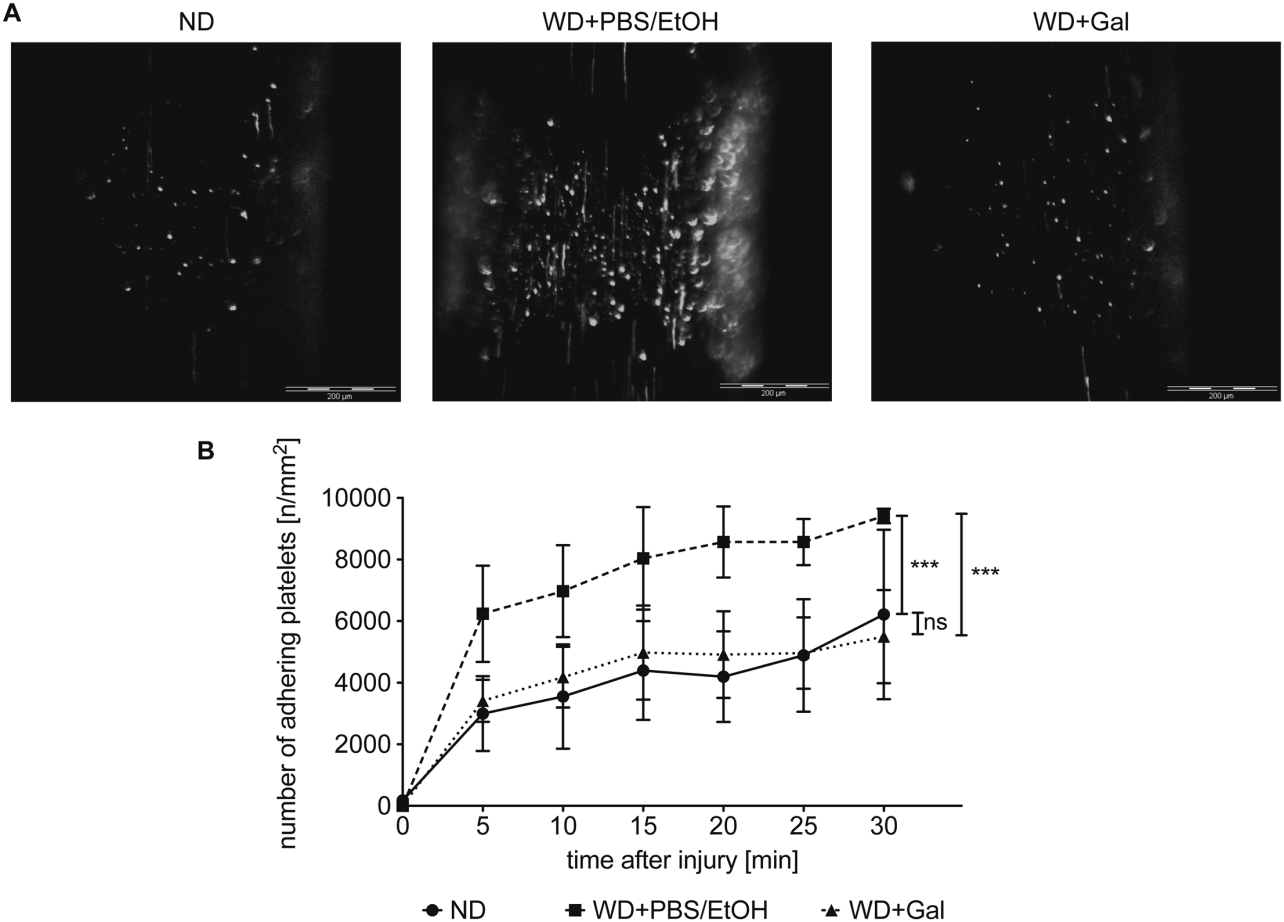
Galiellalactone reduces the adhesion of platelets to the ligation-injured arteria carotis communis. In a common carotid artery thrombosis model, carotid injury in ApoE-deficient mice either fed for 18 weeks with normal chow diet (ND) or western-type diet (WD) (treated with Galiellalactone (Gal) or vehicle (PBS/EtOH) for 6 weeks) was induced for 5 minutes by transient ligation. The adhesion of fluorescence-labeled platelets of donor mice to the injured blood vessel was observed every 5 minutes after injury *in situ* by high-speed intra-vital epifluorescence high-speed video microscopy for an observation period of 30 minutes. The upper panel (A) shows representative images of adhering platelets 30 minutes after injury. Data shown in the lower panel (B) are mean ± SEM of 3–4 mice (*** = p < 0.001 vs. WD + PBS/EtOH; ns = not significant vs. ND; two-way ANOVA).

Feeding the mice with WD for 18 weeks increased the adhesion of platelets to the injured area by two-fold (Fig 3 and S1–S3 movies) when compared to ND mice. Gal-treatment reverted this increase to the level of adherent platelet counts of mice fed with ND.

**Fig 4:**
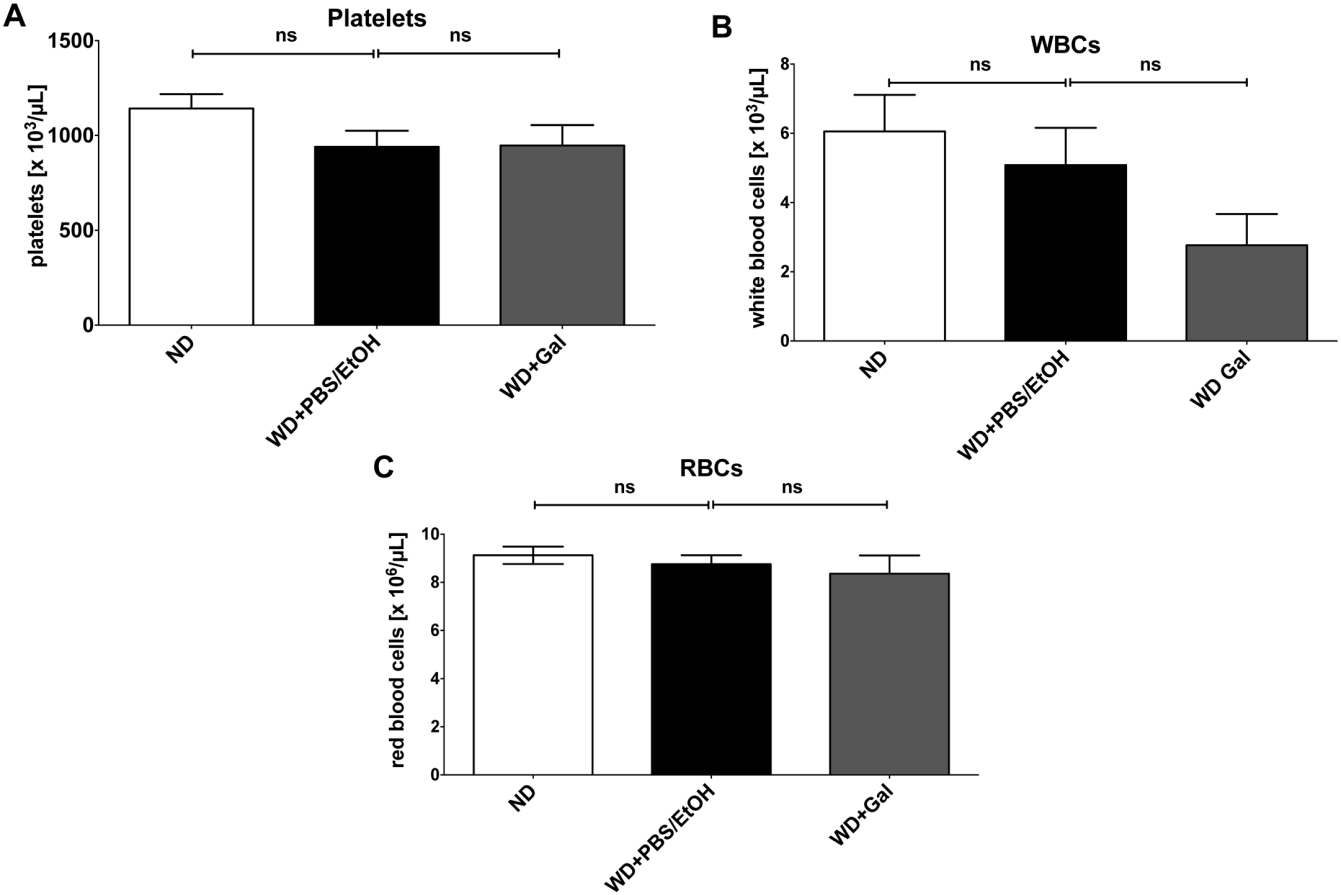
Galiellalactone has no effect on blood cell counts in western type diet-treated ApoE-deficient mice. ApoE-deficient mice were either fed for 18 weeks with normal chow diet (ND) or western-type diet (WD) (treated with Galiellalactone (Gal) or vehicle (PBS/EtOH) for 6 weeks). Citrated whole mouse blood was collected by *intra-cardial* puncture. Platelet counts (**A**), white blood cell counts (WBCs; **B**) as well as red blood cell counts (RBCs; **C**) were determined using an automatic cell counter. Data shown are mean + SEM of 4–6 mice (ns = not significant vs. WD + PBS/EtOH; one-way ANOVA).

To analyze whether Gal changes the numbers of the different blood cells, we performed blood cell counts. As seen in Fig 4, no effects of Gal on the blood cells counts in WD-fed animals were detected.

## Discussion

Increased cardiovascular mortality of patients suffering from inflammatory diseases is related to enhanced atherosclerotic and thrombotic events that are independent of the classic risk factors for cardiovascular death [6, 28]. Elevated inflammation seems to contribute to this enhanced risk [7] and, therefore, new drugs that on the one hand are able to lower inflammation and on the other hand reduce the cardiovascular mortality by preventing arterial thrombus formation are needed.

Murine models with highly elevated pro-inflammatory gene expression (like Tristetraprolin-deficient mice [29]) have a complex disease phenotype and are not well suited for long-term analyses needed for cardiovascular research due to their markedly reduced life span. The cardiovascular phenotype of such mice is generally insufficiently characterized. In contrast, the ApoE-deficient mice are a well-established model of atherosclerosis. Moreover, the atherothrombotic changes in ApoE-deficient mice have been correlated to enhanced inflammation [30, 31]. Therefore, selected this model to study the anti-inflammatory and anti-thrombotic capacity of the fungal compound Gal [12]. Gal-treatment led to a reduction of WD-induced mRNA expression of different inflammatory and atherosclerotic marker genes (Fig 1). Those results indicate that Gal is able to lower at least the mRNA expression of genes that are involved in atherogenesis and inflammation. As shown in suppl. Fig 1, we found no induction of IL1β or CCL2/MCP1 mRNA expression in the aortas of animals fed with WD compared to ND-treated animals. Contradictory data exist that show either an induction or no induction of IL1β and CCL2 expression by fat containing diets. In a gene expression profiling study by Castro et al [32] no enhanced IL1β or CCL2/MCP1 expression in the aorta of WD-fed ApoE-ko mice were found. In contrast another type of high fat atherogenic diet increased IL1β mRNA expression in the aortas of ApoE-ko mice [33]. In our study WD did not induce IL1β or CCL2/MCP1 mRNA expression in the aorta of ApoE-ko mice. The inability of Gal to reduce IL1β and CCL2/MCP1 mRNA expression in those aortas seems to be mainly contributed to an absence of mRNA-inducibility of these genes by WD.

Coagulation activation and adhesion of platelets to the injured vessel wall are critical mechanisms for the progression of atherosclerosis and thrombosis [34, 35]. Treating atherosclerotic ApoE-deficient mice with Gal significantly prolonged the blood clotting times of plasma of those mice in comparison to vehicle-treated mice (Fig 2). Enhanced blood clotting times may lower the thrombotic risk of the Gal-treated mice, as indicated in the applied platelet adhesion model at the ligation injured common carotid artery (Fig 3). Induction of the atherosclerotic phenotype by WD led to increased adhesion of platelets to the injured vessel wall that could be restored by Gal-treatment to the level of control mice (fed with ND). These changes are not related to Gal-mediated changes in the blood cell counts as shown in Fig 4.

Pro-inflammatory cytokines (e.g. IL1β, IL6, and TNFα), which are systemically enhanced in chronic inflammatory diseases like RA are described to trigger endothelial cells to change their anti-thrombotic properties into a procoagulant state [36]. Gal seems to be able to reduce blood clotting and platelet adhesion to the vessel wall by reduction of the inflammatory burden due to its ability to interfere with different pro-inflammatory signaling pathways (like NF-κB- or STAT-dependent gene expression).

In summary, the results of our study, using the fungal compound Gal for the first time in an atherosclerotic mouse model, showed that Gal is able to lower the expression of an inflammatory cytokine profile and improves key parameters of arterial thrombosis (blood clotting times, platelet adhesion to the ligation injured carotid artery). Therefore, Gal could be a promising new substance or lead-structure for the development anti-atherothrombotic drugs.

## Supporting Information

S1 Fig Western-type diet has no effect on IL1β or CCL2/MCP1 mRNA expression in the aorta of ApoE-deficient mice. RNA isolated from aortas of ApoE-deficient mice either fed for 18 weeks with normal chow diet (ND) or western-type diet (WD) (treated with Galiellalactone (Gal) or vehicle (PBS/EtOH) for 6 weeks) was analyzed for IL1β (**A**) and CCL2/MCP1 (**B**) mRNA expression in qPCR experiments. Data shown are mean + SEM of 4–10 mice (ns = not significant vs. WD + PBS/EtOH; one-way ANOVA).
(TIFF)

S2 Fig Galiellalactone has no effect on the expression of blood clotting factors in the liver of western type diet-treated ApoE-deficient mice. RNA isolated from livers of ApoE-deficient mice either fed for 18 weeks with normal chow diet (ND) or western-type diet (WD) (treated with Galiellalactone (Gal) or vehicle (PBS/EtOH) for 6 weeks) was analyzed for factor II (**FII**; **A**), factor VIII (**FVIII**; **B**), and factor X (**FX**; **C**) mRNA expression in qPCR experiments. Data shown are mean + SEM of 4–10 mice (* = p < 0.05; ns = not significant vs. WD + PBS/EtOH; one-way ANOVA).
(TIFF)

S1 Movie Adhesion of platelets to injured vessel wall in ND mice. Imaging of 5-(and 6-) Carboxyflourescein diacetate succinimidyl ester stained adhering platelets (green) to the injured vessel wall of the common carotid artery in ApoE-deficient mice fed with normal chow diet (ND) 30 min after transient ligature, movie of a representative sample.
(AVI)

S2 Movie Adhesion of platelets to injured vessel wall in WD mice treated with vehicle. Imaging of 5-(and 6-) Carboxyflourescein diacetate succinimidyl ester stained adhering platelets (green) to the injured vessel wall of the common carotid artery in ApoE-deficient mice fed with western-type diet (WD) and treated with vehicle (PBS + 10% ethanol) 30 min after transient ligature, movie of a representative sample.
(AVI)

S3 Movie Adhesion of platelets to injured vessel wall in WD mice treated with Gal. Imaging of 5-(and 6-) Carboxyflourescein diacetate succinimidyl ester stained adhering platelets (green) to the injured vessel wall of the common carotid artery in ApoE-deficient mice fed with western-type diet (WD) and treated with the fungal metabolite Galiellalactone (Gal; 10 mg/kg) 30 min after transient ligature, movie of a representative sample.
(AVI)
